# Assessing and Overcoming Resistance Phenomena against a Genetically Modified Vaccinia Virus in Selected Cancer Cell Lines

**DOI:** 10.3390/ijms21207618

**Published:** 2020-10-15

**Authors:** Susanne Berchtold, Julia Beil, Christian Raff, Irina Smirnow, Martina Schell, Janina D’Alvise, Silvia Gross, Ulrich M. Lauer

**Affiliations:** 1 Department of Internal Medicine VIII, University Hospital Tübingen, D-72076 Tübingen, Germany; susanne.berchtold@uni-tuebingen.de (S.B.); julia.beil@klinikum.uni-tuebingen.de (J.B.); christian.raff@googlemail.com (C.R.); irina.smirnow@klinikum.uni-tuebingen.de (I.S.); martina.schell15@gmail.com (M.S.); Silvia.gross@health.qld.gov.au (S.G.); 2 Cluster of Excellence iFIT (EXC 2180) “Image-Guided and Functionally Instructed Tumor Therapies”, University of Tübingen, D-72076 Tübingen, Germany; 3 German Cancer Consortium (DKTK), German Cancer Research Center (DKFZ), D-72076 Tübingen, Germany; 4 Core Facility for Medical Bioanalytics, Institute for Ophthalmic Research, University Hospital Tübingen, D-72076 Tübingen, Germany; janina.dalvise@uni-tuebingen.de

**Keywords:** oncolytic virotherapy, virotherapy resistance, vaccinia virus, NCI-60 tumor cell panel, super cytosine deaminase prodrug system, chemovirotherapy, 5-fluorocytosine

## Abstract

Genetically modified vaccinia viruses (VACVs) have been shown to possess profound oncolytic capabilities. However, tumor cell resistance to VACVs may endanger broad clinical success. Using cell mass assays, viral replication studies, and fluorescence microscopy, we investigated primary resistance phenomena of cell lines of the NCI-60 tumor cell panel to GLV-1h94, a derivative of the *Lister* strain of VACV, which encodes the enzyme super cytosine deaminase (SCD) that converts the prodrug 5-fluorocytosine (5-FC) into the chemotherapeutic compound 5-fluorouracil (5-FU). After treatment with GLV-1h94 alone, only half of the cell lines were defined as highly susceptible to GLV-1h94-induced oncolysis. When adding 5-FC, 85% of the cell lines became highly susceptible to combinatorial treatment; none of the tested tumor cell lines exhibited a “high-grade resistance” pattern. Detailed investigation of the SCD prodrug system suggested that the cytotoxic effect of converted 5-FU is directed either against the cells or against the virus particles, depending on the balance between cell line-specific susceptibility to GLV-1h94-induced oncolysis and 5-FU sensitivity. The data provided by this work underline that cellular resistance against VACV-based virotherapy can be overcome by virus-encoded prodrug systems. Phase I/II clinical trials are recommended to further elucidate the enormous potential of this combination therapy.

## 1. Introduction

Cancer therapeutic resistance occurs as cancers develop resistance to treatments, such as chemotherapy, radiotherapy, and targeted therapies, through many different mechanisms. These include specific genetic and epigenetic changes in the cancer cell and/or the microenvironment in which the cancer cell resides. Beyond this, current standard therapies are also often burdened with a large number of serious side effects and organ damage. The main reason for this is the inability of these regimes to distinguish healthy tissue from tumor tissue. In contrast, novel biological therapeutics, such as oncolytic viruses, have exactly this capability to exert tumoricidal effects while sparing normal cells/tissues, e.g., by employing a unique primary oncolytic and secondary immunoinductive mechanism of action.

Oncolytic viruses (OVs) mediate their anti-tumoral effect both via a direct and an indirect mechanism of action. They selectively infect and replicate in tumor cells. This massive replication finally leads to a metabolic breakdown of the cell and subsequently to cell lysis, resulting in the release of progeny virions which then can infect hitherto uninfected neighboring cells. In addition, OV-mediated cell death releases cytokines, tumor-associated antigens (TAAs), damage-associated molecular pattern molecules (DAMPs), and pathogen-associated molecular pattern molecules (PAMPs). These are taken up by antigen-presenting cells (APCs); in this course, CD4+ and CD8+ T cells are primed by cross-presentation. This activation of the adaptive immune system then also leads to an anti-tumoral immune response at tumor sites which have not been treated with virus [1,2].

A variety of different virus strains (e.g., adenovirus (AD), reovirus (REO), newcastle disease virus (NDV), herpes simplex virus (HSV), measles vaccine virus (MeV), and vaccinia virus (VACV)) are currently under intensive investigation not only in preclinical but also in clinical studies [3]. Meanwhile, it is assumed that monotherapy with oncolytic viruses might be insufficient to address the sophisticated defense strategies of various tumor types adequately [4,5]. One example are recombinant VACVs which hold great promise as immunotherapeutics and encouraging results could be obtained from basic and translational studies in animal models. However, clinical trials investigating VACVs alone as cancer vaccines have yielded largely disappointing results. Major advances have recently been made to generate recombinant VACVs and other poxviruses to improve their utility as immunotherapeutics [6]. One innovative approach is the integration of suicide genes into the genome of oncolytic viruses, which allows combined cancer treatment with virotherapy and tumor-restricted chemotherapy. One example is the modified vaccinia virus *Ankara* (MVA) containing the yeast-originated transgene *fcu1* (MVA-FCU1), expressing cytosine deaminase and uracil phosphoribosyltransferase enzymes, also called super cytosine deaminase (SCD), that transform the prodrug 5-fluorocytosine (5-FC) into cytotoxic 5-fluorouracil (5-FU) and 5-fluorouridine-5′-monophosphate, respectively [7]. In a first-in-human study, MVA-FCU1 was injected intratumorally (i.t.) in combination with intravenous (i.v.) or oral 5-FC in patients with primary or metastatic liver cancer. It could be shown that the combined treatment strategy was feasible and well tolerated and stable disease could be observed in eight out of 16 patients. It is important to underline that MVA-FCU1 is a non-replicating and therefore non-oncolytic vaccinia virus and that an initial tumor cell infection is the only way to ensure the conversion of 5-FC to 5-FU and the resulting tumor cytotoxicity [8].

Numerous preclinical and clinical studies have already confirmed the efficacy and safety of viral therapeutics with integrated cytosine deaminase-based prodrug-converting systems [9,10,11,12,13]. However, the exact roles and the interplay between primary resistance phenomena to virotherapy and the 5-FU sensitivity of individual tumor cell lines, as well as their connection with the cytotoxic effect of converted 5-FU have not been fully clarified yet. Interestingly, Foloppe and colleagues made the observation that the addition of 5-FC to cultured colon cancer cells followed by infection with vaccinia virus (VV, *Copenhagen* strain) expressing the suicide gene *fcu1* (VV-FCU1) can decrease the level of progeny virus particles. However, this inhibition of virus replication by 5-FC has no negative impact on the anti-tumor efficacy in diverse tumor cell lines as well as in a subcutaneous colon cancer mouse model [13].

Encouraged by the promising data of the first-in-human study with MVA-FCU1, the prodrug-converting system SCD was transferred into a vaccinia *Lister* derivative, yielding a replicating and thus oncolytic strain of vaccinia virus (GLV-1h94). Our rationale was to characterize GLV-1h94 for the treatment of solid tumors, which is why we investigated GLV-1h94 as monotherapy as well as in combination with 5-FC in 54 cell lines of the NCI-60 cell panel representing solid tumors. The main focus addresses the function and efficacy of virus-encoded suicide protein SCD. Moreover, primary resistance phenomena against virotherapy alone and the possibility to overcome this resistance with additional tumor-restricted/local chemotherapy were investigated.

Briefly, we could show that solid NCI-60 tumor cell lines responded with different levels of cellular resistance to GLV-1h94-based virotherapy. However, this resistance can be overcome by using the virus-encoded SCD prodrug system. Detailed investigation of the prodrug system revealed that the cytotoxic effect of converted 5-FU is directed either against the cells or against the virus particles, depending on the balance between cell line-specific susceptibility to GLV-1h94-induced oncolysis and 5-FU sensitivity.

## 2. Results and Discussion

### 2.1. Screening of the NCI-60 Tumor Panel for Resistances to Oncolysis with GLV-1h94

First, the oncolytic efficacy of the *fcu1* suicide gene-encoding virotherapeutic vector GLV-1h94 alone (i.e., without addition of the prodrug 5-FC) was assessed in a comprehensive and enlarged setting in 54 adherent cell lines derived from solid tumors of the NCI-60 panel, a well-established cancer cell line panel [14]. Due to great differences in handling, the six leukemia cell lines of the NCI-60 panel were deliberately excluded.

**Figure 1:**
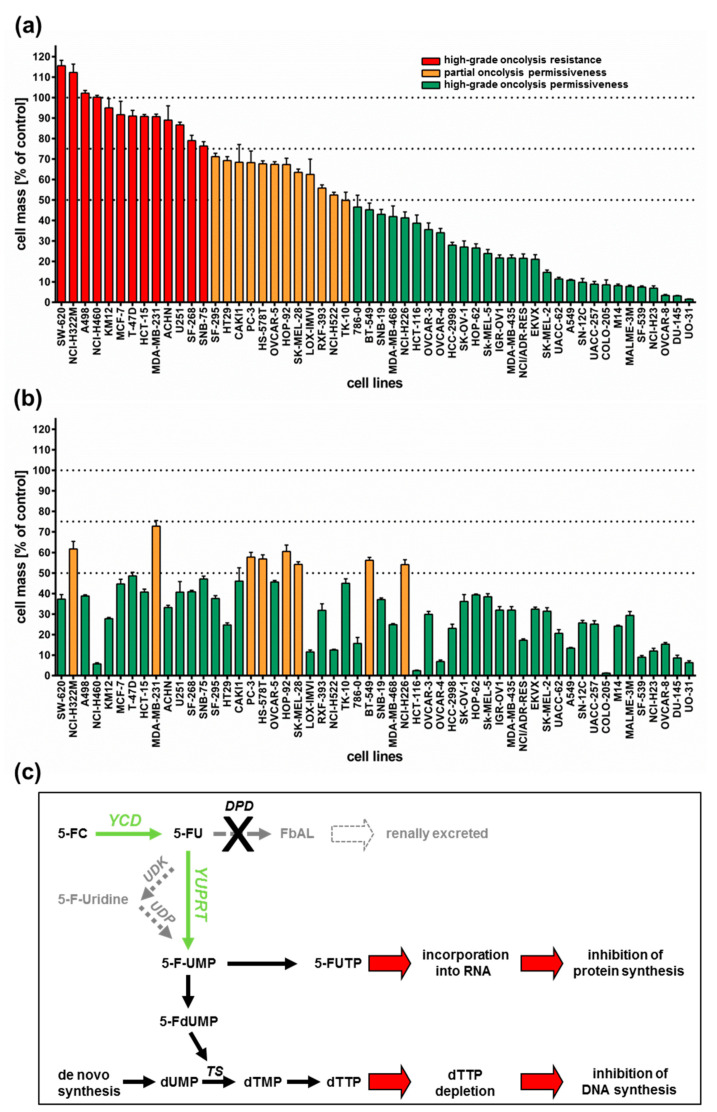
Tumor cell lines of the NCI-60 panel (*n* = 54) infected with the virotherapeutic compound GLV-1h94 without (A) and with (B) addition of the prodrug 5-fluorocytosine (5-FC). (**a**,**b**) At 96 h post infection, tumor cell masses were analyzed via sulforhodamine B (SRB) assay. Bars in red indicate a remnant tumor cell mass of more than 75% (in comparison to mock-infected cells) and thereby define a “high-grade resistance” to GLV-1h94-mediated oncolysis; bars in orange denote remaining tumor cell masses in the range of 50–75%, defining a “partial permissiveness” to oncolysis by GLV-1h94; bars in green specify a remaining tumor cell mass of less than 50%, categorized as “high-grade permissiveness” to GLV-1h94-mediated oncolysis. Mean ± SD of three independent experiments are shown. Thresholds (100%, 75%, 50%) are indicated by dotted lines. (**c**) Overview of the 5-FC conversion system (employing enzymes YCD + YUPRT). dUMP: deoxyuridine monophosphate; TS: thymidylate synthase; dTMP: deoxythymidine mono-phosphate; dTTP: deoxythymidine triphosphate; 5-FdUMP: 5-fluorodeoxyuridine mono-phosphate; 5-FUMP: 5-fluorouridine monophosphate; 5-FUTP: 5-fluorouridine triphosphate; 5-FC: 5-fluorocytosine; YCD: yeast cytosine deaminase; 5-FU: 5-fluorouracil; UDK: uridine kinase; UDP: uridine phosphorylase; YUPRT: yeast uracil phosphoribosyltransferase; DPD: dihydro-pyrimidine dehydrogenase; FbAL: 5-fluoro-ß-alanine. Scheme modified from [15].

All 54 tumor cell lines were infected with GLV-1h94 at MOI 0.1 and tumor cell masses remaining at 96 h post infection (hpi) were determined by a sulforhodamine B (SRB) assay. With regard to the results, three arbitrary response categories were defined (Figure 1a). Tumor cell lines in which the cell mass at 96 hpi decreased by less than 25% (in comparison to mock-infected cells) when using an MOI of 0.1 were termed “high-grade resistant” (depicted in red). Tumor cell lines with a remaining cell mass between 50% and 75% were considered to be “partially permissive” (depicted in orange), whereas tumor cell lines with a remaining cell mass of less than 50% were categorized as “high-grade permissive” (depicted in green).

As a result, more than half of the tested tumor cell lines (54%) turned out to be “high-grade permissive” regarding GLV-1h94-mediated oncolysis. Another 22% of the tested tumor cell lines were considered as “partially permissive” and 24% were found to be “high-grade resistant” (Figure 1a). In each of the eight different tumor entities (breast, central nervous system (CNS), colon, lung, melanoma, ovarian, prostate, and renal cancer), “high-grade permissive”, “partially permissive”, and “high-grade resistant” tumor cell lines could be identified. Interestingly, we did not find any correlation between oncolytic permissiveness and origin/phenotype of the different solid tumor cell lines. However, it was observed that melanoma cell lines in particular reacted very positively to GLV-1h94 infections, seven of nine melanoma cell lines being high-grade permissive. In this context, it is of great interest that an early clinical study already successfully investigated the direct i.t. treatment of malignant melanomas using a wild-type vaccinia virus. In this study, six out of ten patients responded with a complete remission after intralesional (i.l.) virus application [16]. With regard to the current progress of clinical virotherapy, T-VEC, another genetically modified DNA virus, was approved in the US and in Europe in 2015 as the world’s first viral drug (Imlygic^®^) for immunotherapy of patients with unresectable, locally advanced or distant metastatic melanoma [17]. This data correlation gives reason to believe that malignant melanoma represents a prototypic tumor disease that can be effectively treated by virotherapy.

Next, we investigated whether addition of the prodrug 5-fluorocytosine (5-FC) was able to enhance the anti-tumoral efficacy of GLV-1h94, thus exploiting the *fcu1* suicide gene function (an overview of the 5-FC conversion system is depicted in Figure 1c). For this purpose, 5-FC was added at 3 hpi and SRB assays were performed at 96 hpi as described (Figure 1b). Interestingly, all tumor cell lines, which previously were rated as “high-grade resistant” to GLV-1h94 infection now turned out to be “partially permissive” or even “high-grade permissive” tumor cell lines after combined treatment of GLV-1h94 (MOI 0.1) and 5-FC (1 mmol/l) (Figure 1b; please note that all tumor cell lines are depicted in the same order as used before in Figure 1a). In particular, administering the prodrug 5-FC post infection changed the pattern of the 13 formerly “red”/“high-grade resistant” tumor cell lines (Figure 1a) to 11 “high-grade permissive” and two “partially permissive” cell lines (Figure 1b). Furthermore, out of the tumor cell lines previously classified as “partially permissive” (*n* = 12; depicted in yellow in Figure 1a), four stayed “partially permissive” to combined treatment with no change in the remaining tumor cell masses, whereas eight cell lines turned into “high-grade permissive” tumor cell lines. This observation can be explained by the successful intracellular conversion of the prodrug 5-FC to 5-FU, which exerts its cytotoxic effect not only directly on infected cells but also on uninfected neighboring cells via its strong bystander effect [18,19]. Similar results are shown in a recent publication, in which the anti-tumor effect of attenuated vaccinia *Tian Tan* strain Guang 9 (VG9), with active yeast cytosine deaminase (CD) expression and thymidine kinase (TK) deficiency, was evaluated in different cancer cell lines. Wild-type VG9 and VG9-CD both showed an identical oncolytic potential, which, however, was significantly different depending on the treated cell line. Their experiments also showed that the addition of 5-FC to VG9-CD-infected cells increased the oncolytic effect significantly compared to VG9-CD treatments alone. The authors postulate that the synergetic effect was more effective when the VG9-CD titers reached at least a level of MOI 1, indicating that the conversion efficiency of CD is only effective above a certain concentration of the expressed CD protein [20]. In a first-in-human study, a modified vaccinia virus *Ankara* (MVA) containing the yeast-originated transgene *fcu1* (TG4023) was investigated in patients with metastatic liver tumors [8]. The clinical trial aimed to assess the maximum tolerated dose (MTD) of TG4023 and the safety, feasibility, and proof of concept (PoC) of TG4023/5-FC combination to deliver high 5-FU concentrations in tumors. Cancer patients underwent a percutaneous i.t. injection of TG4023 on day 1 using ultrasound guidance followed by i.v. and/or oral 5-FC applications for 14 days. In summary, this phase I study demonstrated that i.t. injection of TG4023 was well tolerated and the MTD was defined as 4 × 10^8^ pfu. Therapeutic 5-FU concentrations could be documented in tumors, indicating the proof of concept of virus-directed prodrug therapy. Furthermore, locally measured 5-FU concentrations were higher in tumor tissues compared to blood 5-FU levels, which might explain the reduced systemic toxicity of this special kind of tumor-restricted chemotherapy [8].

In this study, two of 29 tumor cell lines, which before were classified as “high-grade permissive” to GLV-1h94 monotherapy, now exhibited a “partially permissive” pattern when undergoing combined treatment (see breast cancer cell line BT-549 as well as lung cancer cell line NCI-H226). This phenomenon, on the one hand, might be explained by simple assay variations; on the other hand, it also might be reasonable to believe that converted 5-FU reduces the efficacy of the DNA virus GLV-1h94 due to its function as a thymidylate synthase inhibitor and thus not only prohibits genomic DNA but also viral DNA replication and consequently progeny virus synthesis.

Based on these results, all following experiments were performed exemplarily only with the two “high-grade resistant” tumor cell lines NCI-H460 and HCT-15 as well as with the two “high-grade permissive” cell lines OVCAR-8 and DU-145.

### 2.2. 5-FU Sensitivity Is Cell Line Specific

Based on the results of the NCI-60 tumor cell screening, which found that all tested cell lines reacted to the addition of 5-FC, but individually to varying extents, the 5-FU sensitivity of selected cell lines was investigated. For this assay, the two “high-grade resistant” tumor cell lines NCI-H460 and HCT-15, which showed significant tumor cell reduction with the addition of 5-FC after infection with GLV-1h94 (Figure 1b), and the two “high-grade permissive” tumor cell lines OVCAR-8 and DU-145, cell lines whose permissivity hardly changed due to prodrug activation (Figure 1b), were treated with increasing 5-FU concentrations for 24, 48, 72, and 96 h and cell masses were determined by SRB assays.

**Figure 2:**
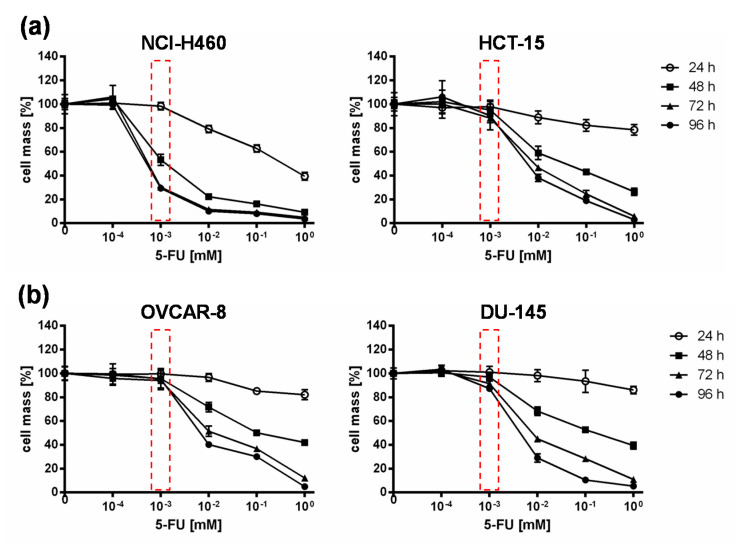
5-fluorouracil (5-FU) sensitivity of two “high-grade resistant” ((**a**); NCI-H460, HCT-15) and two “high-grade permissive” ((**b**); OVCAR-8, DU-145) tumor cell lines. All tumor cell lines were treated with different concentrations of 5-FU (10^−4^, 10^−3^, 10^−2^, 10^−1^, 1 mM) and cell masses were analyzed at 24, 48, 72, and 96 h via SRB assay. Mean ± SD of two independent experiments are shown. Red boxes indicate relevant differences in responsiveness to 5-FU between all cell lines at a concentration of 10^−3^ mM 5-FU at which the greatest difference between the tested cell lines with respect to tumor cell masses was observed.

When comparing all four cell lines, it could be shown that in NCI-H460 cells even small amounts of 5-FU (10^−3^ mM) were sufficient to reduce tumor cells to a remaining cell mass of ~60 % after 48 h and ~30 % (Figure 2) after 96 h. In contrast, higher concentrations of 5-FU were required for the second “high-grade resistant” cell line HCT-15 (Figure 2a) as well as for both “high-grade permissive” cell lines (Figure 2b), to achieve nearly the same cell reduction at both time points. These results indicate a cell line-specific sensitivity against 5-FU. It is known that 5-FU sensitivity is influenced by expression levels of dihydropyrimidine dehydrogenase, the genetic status of p53, and DNA mismatch repair genes [19]. Furthermore, a study from Wang et al. investigated five pairs of 5-FU-resistant and relevant drug-sensitive parental cancer cell lines to unravel specific molecular factors and cellular pathways mediating and/or predicting 5-FU resistance [21]. They could show that 5-FU resistance is multifactorial and involves some or all of the following cellular pathways: overproduction of 5-FU targets, up-regulation of specific anti-apoptotic proteins, reduced production of 5-FU–activating enzymes, and increased G1 checkpoint stringency with a reduced cell proliferation rate and reduction in DNA synthetic machinery [21].

### 2.3. Prodrug Activation Reduces the Replication of Virus Particles in Tumor Cells Regardless of Their Resistance Classification

**Figure 3:**
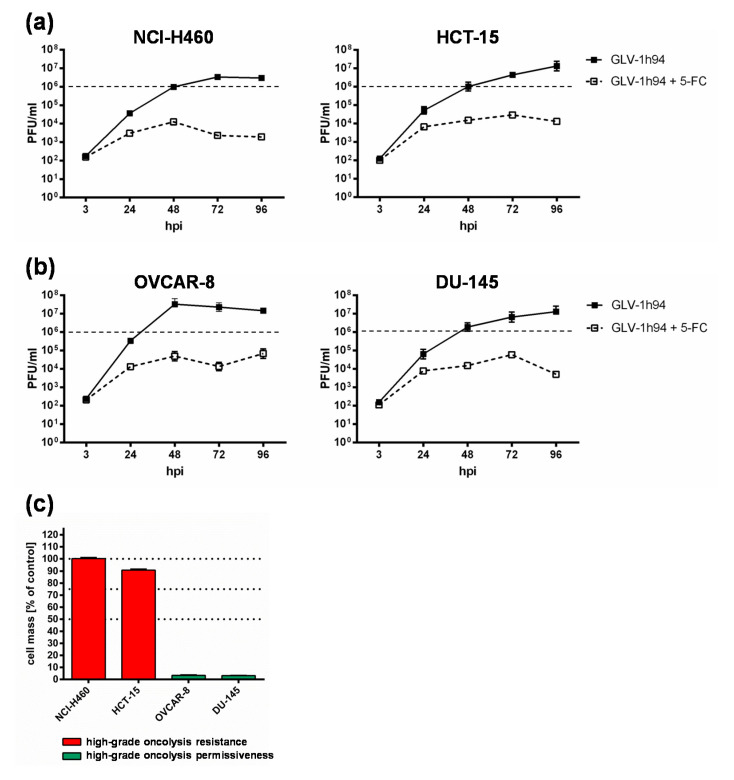
Replication of GLV-1h94 in two “high-grade resistant” ((**a**); NCI-H460, HCT-15) and in two “high-grade permissive” ((**b**); OVCAR-8, DU-145) tumor cell lines alone and after the addition of the prodrug 5-fluorocytosine (5-FC). (**a**,**b**) All four tumor cell lines were infected with GLV-1h94 at MOI 0.1. At 3 hpi, 5-FC [0.1 mM] was added and replication was analyzed via plaque assay at 3, 24, 48, 72, and 96 hpi. (**c**) Remaining cell masses of NCI-H460, HCT-15, OVCAR-8, and DU-145 tumor cells measured via SRB assay at 96 hpi with GLV-1h94 (MOI 0.1). Mean ± SD of two independent experiments are shown. Dashed lines indicate a virus concentration of 10^6^ PFU/mL to better illustrate differences between the analyzed cell lines. PFU: plaque-forming units; MOI: multiplicity of infection; hpi: hours post infection with GLV-1h94.

To get a closer insight into the differences of “high-grade resistant” and “high-grade permissive” tumor cell lines, viral replication was analyzed in the two “high-grade resistant” cell lines NCI-H460 and HCT-15 as well as in the two “high-grade permissive” cell lines OVCAR-8 and DU-145 (Figure 3).

Surprisingly, the differences in viral replication of GLV-1h94 alone without prodrug activation in “high-grade permissive” or “high-grade resistant” tumor cells were not as distinct as expected (Figure 3). However, slightly increased replication tended to occur in OVCAR-8 cells, where viral titers at 48 hpi were more than one log level higher than in the other three cell lines (Figure 3b, left panel). Usually, after tumor cell infection, oncolytic viruses take complete command of the transcription and translation machinery of the virus host cell to produce the largest possible number of progeny virus particles. If the cellular viral load becomes too large, it will lead to oncolysis and, as a result, to a massive release of newly formed infectious virus particles [4].

Of note, clinical correlates of vaccinia virus-induced oncolysis were demonstrated in a recent phase I trial [22]. Cancer patients receiving virus construct GLV-1h68 (closely related to GLV-1h94) exhibited a profound replication of GLV-1h68, which resulted in a distinctive oncolysis, as demonstrated by the release of the GLV-1h68-encoded ß-glucuronidase marker protein as well as of the cell-based enzyme lactate dehydrogenase (LDH), which both became detectable in diverse body fluids (e.g., in the patients´ plasma). Thus, clinical evidence of a substantial vaccinia virus-induced oncolysis was provided.

In consequence, substantially higher titers would be expected in the “high-grade permissive” cancer cell lines and in this study the “high-grade resistance” phenomenon of NCI-H460 and HCT-15 cells against GLV-1h94-mediated oncolysis cannot be explained by a reduced viral replication in these cell lines. On the contrary, in a preclinical study with an oncolytic vesicular stomatitis virus (VSV), it was shown that tumor regression of B16 melanomas was not associated with progressive rounds of virus replication and subsequent oncolysis but rather correlated with viral gene expression and the induction of pro-inflammatory reactions in this tumor model [23].

When investigating GLV-1h94 replication after prodrug activation by the addition of 5-FC, it became noticeable that in all tested cell lines, the formation of progeny virus particles was massively impaired compared to GLV-1h94 replication alone over the entire observation period of 96 hpi (Figure 3a,b). Accordingly, prodrug activation led to a reduction of viral replication by approximately three log levels independent of the resistance classification of the cell line used. In addition, it could be shown that, in particular, the cell line NCI-H460, which was classified as “high-grade resistant” to GLV-1h94 monotherapy, became “high-grade permissive” after activation of the prodrug system (Figure 1a,b), although viral replication was severely restricted. This indicates that cell death does not solely depend on viral replication and oncolysis but that the cytotoxic effect of 5-FU, which is formed by virus-produced SCD from 5-FC, plays a major role. Two scenarios are conceivable in this context; either 5-FU reduces the replication of the DNA virus GLV-1h94 due to its function as a thymidylate synthase inhibitor and thus not only inhibits genomic DNA but also viral DNA replication and consequently progeny virus synthesis [19] or it also might be reasonable that 5-FU blocks the proliferation of tumor cells which is an important prerequisite for productive virus replication [24]. To what extent each of the postulated scenarios apply and whether there is a possible additional dependence on the cell line-specific resistance classification and the 5-FU sensitivity must be elucidated in further experiments.

### 2.4. Conversion Rate of 5-FC into 5-FU Depends on the Cell Line-Specific Infection Rate of GLV-1h94

**Figure 4:**
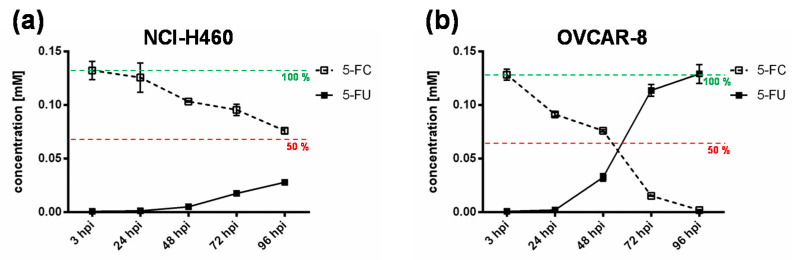
Metabolic conversion of 5-fluorocytosine (5-FC) into 5-fluorouracil (5-FU) after infection of a “high-grade resistant” ((**a**); NCI-H460) and a “high-grade permissive” ((**b**); OVCAR-8) tumor cell line. Both tumor cell lines were infected with GLV-1h94 at MOI 0.01. At 3 hpi, 5-FC [0.1 mM] was added and supernatants were collected at 3, 24, 48, 72, and 96 hpi. Concentrations of 5-FC and 5-FU were determined by mass spectrometry. MOI: multiplicity of infection; hpi: hours post infection with GLV-1h94.

To further elucidate the impact of the chemotherapeutic compound 5-FU, the metabolic conversion rate of 5-fluorocytosine (5-FC) into 5-fluorouracil (5-FU) after infection of the “high-grade resistant” cell line NCI-H460 and the “high-grade permissive” cell line OVCAR-8 with GLV-1h94 and the addition of 5-FC was analyzed by mass spectrometry (Figure 4). Here, we deliberately investigated solely the cell lines NCI-H460 and OVCAR-8, because the metabolic conversion rate is only interesting for cell lines that show strong differences in 5-FU sensitivity, which is not the case for HCT-15 and DU-145.

As expected, the conversion rates in both cell lines differed greatly from each other. Accordingly, the conversion rate in NCI-H460 cells at 96 hpi was below 50% (Figure 4a), whereas, at the same time, a conversion rate of 100% was detected in OVCAR-8 cells (Figure 4b). These findings correlate directly with the results of the infection and cell mass studies of the NCI-60 panel (Figure 1a, b) as well as with the 5-FU sensitivity testing (Figure 2). The cell line NCI-H460 displayed a very high resistance to GLV-1h94-mediated oncolysis with nearly 100% remaining cell mass after infection with GLV-1h94 alone at 96 hpi (Figure 1a). Therefore, it can be assumed that only a few cells are infected and only a small amount of SCD is produced. This lack of prodrug-converting enzyme in turn means that little 5-FC can be converted to 5-FU, which explains the conversion rate of less than 50% (Figure 4a). However, NCI-H460 cells responded very efficiently to the combination of viro- and chemotherapy with a remaining cell mass of 5% after prodrug activation (Figure 1b). This effect can be explained by the cell line’s very pronounced sensitivity to 5-FU (Figure 2), indicating that the conversion rate of 5-FC to 5-FU is still high enough to result in massive chemotoxic tumor cell lysis. In contrast, OVCAR-8 is a “high-grade permissive” cell line (3% remaining cell mass 96 hpi with GLV-1h94; Figure 1a), which is why a large number of cells are initially infected and, therefore, a large amount of prodrug-converting enzyme is expressed in the cells. Hence, the added 5-FC was completely converted into 5-FU and a conversion rate of 100% (Figure 4b) was measured, which confirms that the conversion rate depends on the cell line-specific infection rate of GLV-1h94. Since a large number of cells are already initially infected and OVCAR-8 cells are less sensitive to 5-FU, the large amount of converted 5-FU does not cause an additional cytotoxic effect. It rather seems that in this context 5-FU has a negative influence on viral replication due to its function as a thymidylate synthase inhibitor, which would explain the slight increase in cell mass 96 hpi after combination therapy (15% remaining cell mass; Figure 1b).

These complex correlations indicate that the chemotoxic effect of converted 5-FU is directed against the tumor cells as well as against the virus particles. The balance between cell line-specific susceptibility to GLV-1h94-induced oncolysis and 5-FU sensitivity determines which effect predominates.

### 2.5. Visualization of the Different Targets of 5-FU by Fluorescence Microscopy

**Figure 5:**
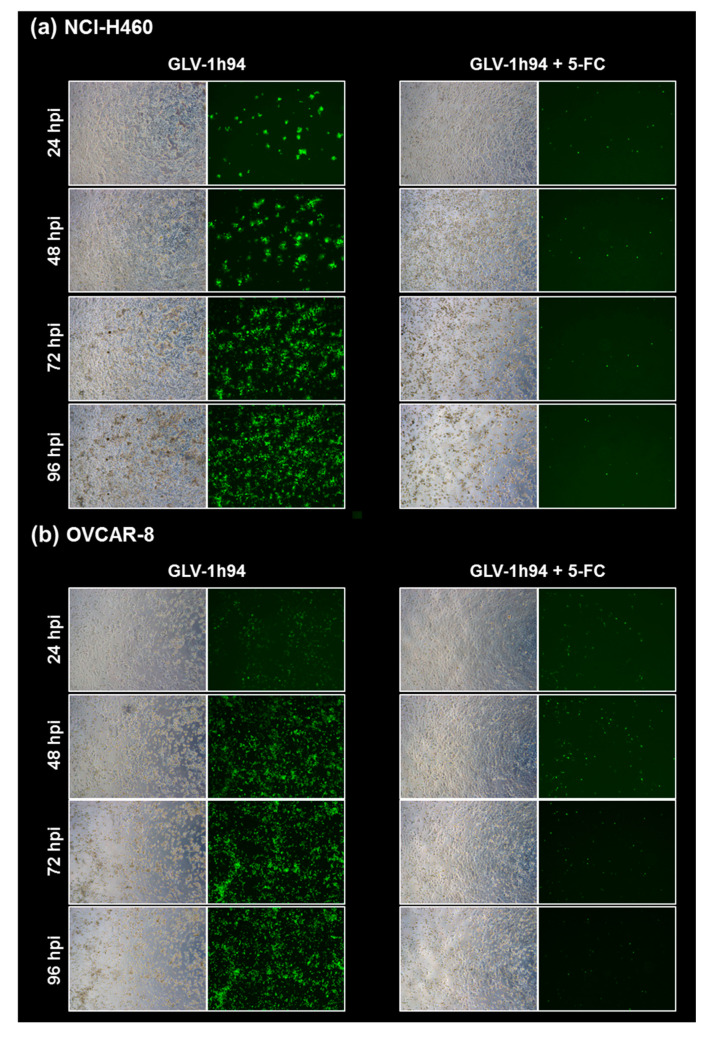
Fluorescence images of the “high-grade resistant” tumor cell line NCI-H460 (**a**) and the “high-grade permissive” tumor cell line OVCAR-8 (**b**) infected with GLV-1h94 ± prodrug 5-fluorocytosine (5-FC). Both tumor cell lines were infected with GLV-1h94 at MOI 0.1. At 3 hpi, 5-FC [0.1 mM] was added and brightfield (left panels) as well as fluorescence images (right panels) were taken at 24, 48, 72, and 96 hpi. Images were taken with a Leica DMi8 microscope equipped with a DMC 4500 camera, original magnification 50×.

**Figure 6:**
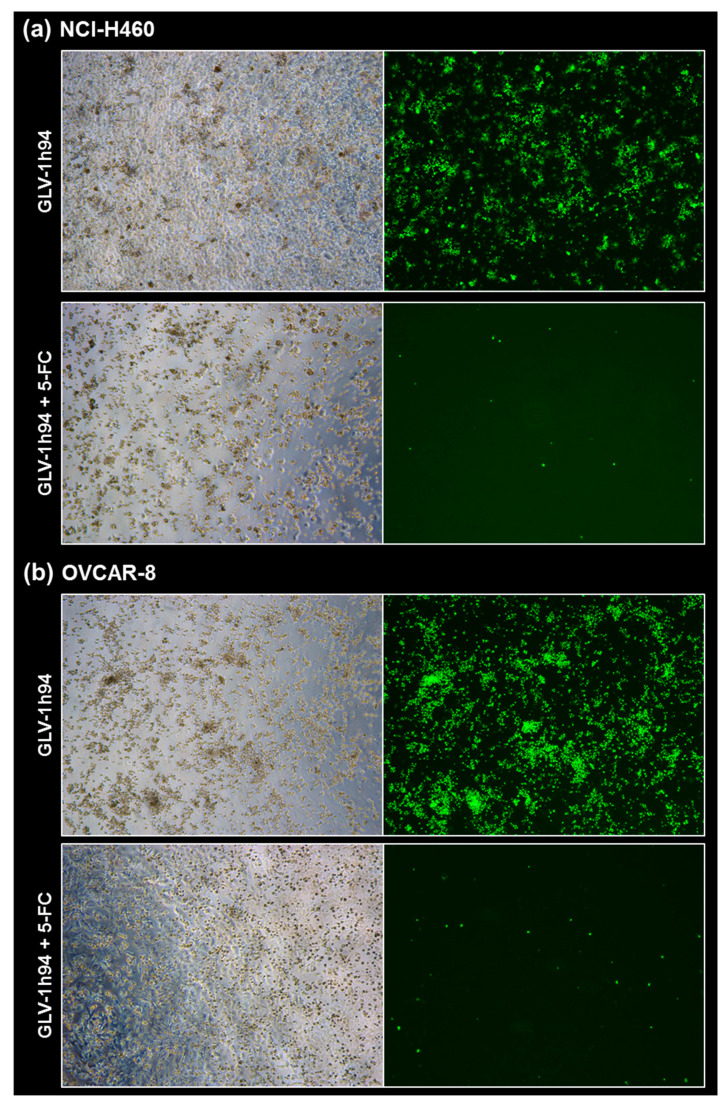
Fluorescence images of the “high-grade resistant” tumor cell line NCI-H460 (**a**) and the “high-grade permissive” tumor cell line OVCAR-8 (**b**) infected with GLV-1h94 ± prodrug 5-fluorocytosine (5-FC). Both tumor cell lines were infected with GLV-1h94 at MOI 0.1. At 3 hpi, 5-FC [0.1 mM] was added and brightfield (left panels) as well as fluorescence images (right panels) were taken at 96 hpi. Images were taken with a Leica DMi8 microscope equipped with a DMC 4500 camera, original magnification 50×.

**Figure 7:**
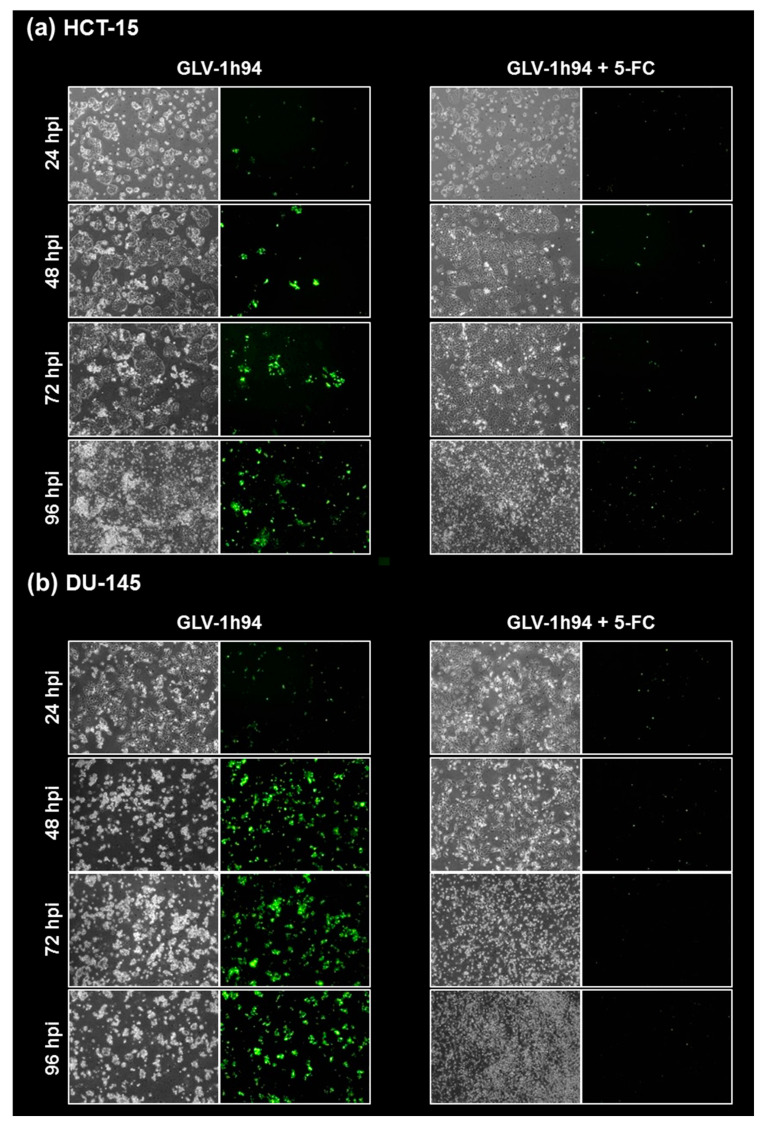
Fluorescence images of the “high-grade resistant” tumor cell line HCT-15 (**a**) and the “high-grade permissive” tumor cell line DU-145 (**b**) infected with GLV-1h94 ± prodrug 5-fluorocytosine (5-FC). Both tumor cell lines were infected with GLV-1h94 at MOI 0.1. At 3 hpi, 5-FC [0.1 mM] was added and brightfield (left panels) as well as fluorescence images (right panels) were taken at 24, 48, 72, and 96 hpi. Images were taken with an Olympus IX50 microscope equipped with an F-view Soft Imaging System camera, original magnification 40×.

**Figure 8:**
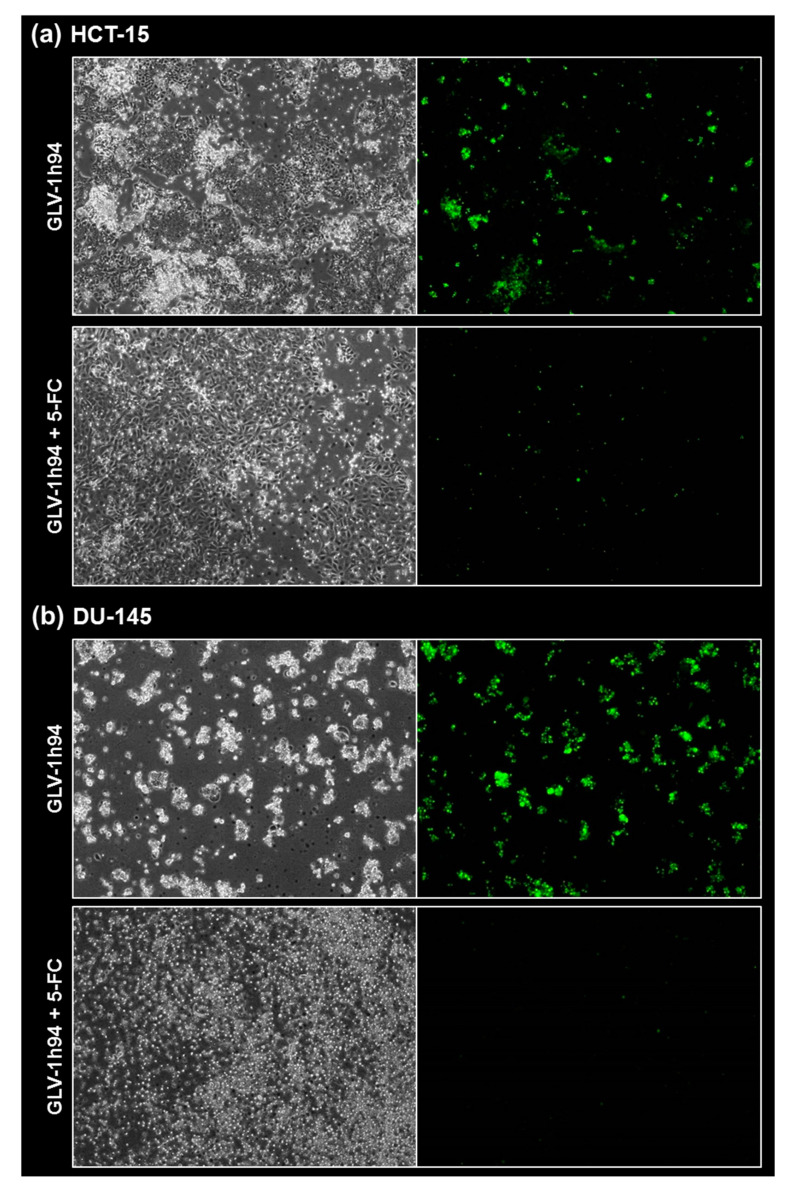
Fluorescence images of the “high-grade resistant” tumor cell line HCT-15 (**a**) and the “high-grade permissive” tumor cell line DU-145 (**b**) infected with GLV-1h94 ± prodrug 5-fluorocytosine (5-FC). Both tumor cell lines were infected with GLV-1h94 at MOI 0.1. At 3 hpi, 5-FC [0.1 mM] was added and brightfield (left panels) as well as fluorescence images (right panels) were taken at 96 hpi. Images were taken with an Olympus IX50 microscope equipped with an F-view Soft Imaging System camera, original magnification 40×.

To confirm and, more importantly, visualize the developed hypotheses, fluorescence and brightfield images of NCI-H460 and OVCAR-8 (Figure 5 and Figure 6) as well as of HCT-15 and DU-145 cells (Figure 7 and Figure 8) were taken at 24, 48, 72, and 96 hpi or solely at 96 hpi after GLV-1h94 monotherapy as well as after prodrug activation by the addition of 5-FC. Since the viral construct GLV-1h94 encodes the marker protein GFP, it is possible to observe virus infection by fluorescence microscopy. In the “high-grade resistant” cell lines NCI-H460 (Figure 5a, left panel) and HCT-15 (Figure 7a, left panel), infection with GLV-1h94 alone could be proven by detection of GFP expression starting at 24 hpi and increasing until 96 hpi. However, when looking at the cell layer in the corresponding brightfield images, in both cell lines, almost no oncolysis could be detected over the course of infection (Figure 5a and Figure 6a, upper panel). This result confirms that both NCI-H460 and HCT-15 are “high-grade resistant” cell lines, which indeed allow infection with GLV-1h94 and corresponding protein expression to a restricted extent but do not respond to virus replication with oncolysis. Interestingly, when activating the prodrug system by adding 5-FC at 3 hpi, no GFP expression could be observed in NCI-H460 cells as early as 24 hpi and throughout the entire course of infection (Figure 5a, right panel), indicating a reduced viral infection. However, when looking at the brightfield images of the NCI-H460 cell layers, especially at 96 hpi (Figure 6a, lower panel), it is noticeable that their cell mass was strongly reduced, also confirmed by SRB analysis which revealed a cell mass reduction of ~95 % (Figure 1b). Based on these results, one could speculate that in this specific cell line, which is very sensitive to 5-FU (Figure 2), converted 5-FU inhibits cell proliferation itself, which indirectly also stops viral replication, rather than directly inhibits virus replication. Remarkably, when focusing on the brightfield images of the HCT-15 cell layers after prodrug activation over the time course of 96 hpi (Figure 7a, right panel), and particularly at 96 hpi (Figure 8a, lower panel), it is noticeable that their cell mass was only reduced to a small extent. This result indicates that, specifically in HCT-15 cells, which have a reduced 5-FU sensitivity compared to NCI-H460, converted 5-FU partially inhibits both cell proliferation and virus replication, resulting in a “combined” cell mass reduction of only about 57 % (Figure 1b).

When observing the “high-grade permissive” cell lines OVCAR-8 (Figure 5b, left panel) and DU-145 (Figure 7b, left panel), it was found that infection with GLV-1h94 alone leads in both cell lines to a massive GFP expression starting at 24 hpi which increases until 96 hpi. In addition, a progressive destruction of both OVCAR-8 and DU-145 cell layers could be observed (Figure 5b and Figure 7b, left panel). These microscopic images, especially the close-ups of the 96 hpi time points (Figure 6b, upper panel; Figure 8b, upper panel), confirm that both cell lines are “high-grade permissive” cell lines, in which an increase in GLV-1h94 virus replication also led to efficient oncolysis. Surprisingly, when adding 5-FC to the infected cells, no GFP expression and, therefore, reduced viral infection could be observed at 24 hpi and throughout the entire course of infection as in NCI-H460 cells (Figure 5b and Figure 7b, right panel). Interestingly, when looking at the close-ups of the 96 hpi time points, both the OVCAR-8 as well as the DU-145 cell layers were significantly less destroyed than after GLV-1h94 monotherapy (Figure 6b and Figure 8b, lower panel). These results suggest that in both “high-grade permissive” cell lines, compared to the “high-grade resistant” cell line NCI-H460, the primary infection rate is high, resulting in high amounts of prodrug convertase. Therefore, added 5-FC is completely converted to 5-FU, thus achieving high levels that could actually inhibit cell proliferation. However, both OVCAR-8 and DU-145 are cell lines with low 5-FU sensitivity, so it can be speculated that the high 5-FU concentrations in these specific cell lines directly inhibit virus replication (as reflected by a lack of GFP expression) rather than cell proliferation.

In conclusion, the microscopic analysis of GLV-1h94 infection either as monotherapy or with prodrug activation as combined virochemotherapy confirms that the cytotoxic effect of converted 5-FU indeed depends on the balance between cell line-specific susceptibility to GLV-1h94-induced oncolysis and cell line-inherent 5-FU sensitivity. Thus, in a cell line like OVCAR-8 or DU-145, which are permissive to GLV-1h94 infection and at the same time less sensitive to 5-FU, the cytostatic effect is more likely to be directed towards direct virus replication. If, however, a cell line is more resistant to GLV-1h94, like NCI-H460, but shows high 5-FU sensitivity, converted 5-FU (now in lower amounts) tends to inhibit cell proliferation and thus indirectly virus replication, as the breeding ground for the production of progeny virus particles is missing.

## 3. Materials and Methods

### 3.1. Cell Lines

The US National Cancer Institute’s NCI-60 tumor cell panel was purchased from Charles River Laboratories (Charles River Laboratories Inc., New York, NY, USA). African green monkey kidney fibroblasts (CV-1 cells) were purchased from ATCC^®^ (CCL-70TM) (Manassas, VA, USA). All cell lines were grown in Dulbecco’s modified Eagle’s medium (DMEM, Biochrom, Berlin, Germany) supplemented with 10% fetal calf serum (FCS, Sigma-Aldrich, Taufkirchen, Germany). Cells were cultivated at 37 °C in a humidified atmosphere containing 5% CO_2_. Mycoplasma testing of all cell lines was performed regularly every three months (MycoTOOL PCR Mycoplasma Detection Kit, Roche, Mannheim, Germany).

### 3.2. Virus

**Figure 9:**
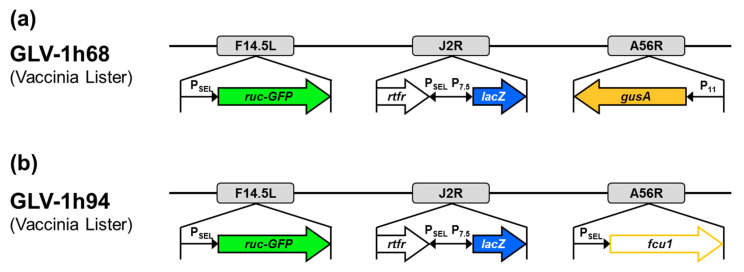
Vaccinia virus constructs GLV-1h68 (**a**) and GLV-1h94 (**b**). *F14.5L* encodes a short polypeptide of 49 amino acids, which was found to be highly conserved among different vaccinia strains, as well as other poxviruses; *J2R*, gene locus for thymidine kinase; *A56R*, gene locus for hemagglutinin; P_SEL_, vaccinia synthetic early/late promoter; P_7.5_, vaccinia early late promoter; P_11_, vaccinia late promoter; *ruc-GFP*, *Renilla* luciferase and *Aequorea* green fluorescent protein; *rtfr*, genes for human transferrin receptor; *lacZ*, ß-galactosidase; *gusA*, ß-glucuronidase; *fcu1*, yeast-originated transgene-expressing cytosine deaminase and uracil phosphoribosyltransferase.

In this study, the vaccinia virus construct GLV-1h94, which was kindly provided by Genelux Corporation, San Diego, CA, USA, was used. GLV-1h94 (Figure 9b) is a derivative of the previously described virus construct GLV-1h68 (Figure 9a) [25]. Both constructs were originated from the *Lister* strain of vaccinia and were generated by homologous recombination of foreign genes into target loci of the VACV genome through double reciprocal crossover. In GLV-1h94, the gene locus for *F14.5L* was deleted by insertion of the fusion protein consisting of *Renilla* luciferase and *Aequorea* green fluorescent protein (ruc-GFP) under control of a vaccinia synthetic early/late promoter (P_SEL_). Furthermore, the gene locus for thymidine kinase (*J2R*) was deleted by insertion of ß-galactosidase (*lacZ*; under control of the vaccinia early late promoter (P_7.5_)) and genes for human transferrin receptor (*TFR*; under control of the synthetic P_SEL_ promoter), which allows for the detection of virus-infected cells at the protein level. Finally, the gene cassette *A56R* (encoding for hemagglutinin), which was deleted by insertion of ß-glucuronidase (*gusA*) in GLV-1h68, was replaced by the suicide gene *fcu1* under control of P_SEL_. The expression of the converting enzyme SCD enables the transformation of 5-FC into 5-FU, which leads to RNA miscoding and direct inhibition of DNA synthesis.

GLV-1h94 was propagated in CV-1 cells. Cells were infected with GLV-1h94 at MOI 0.05. At 48 hpi, cells were harvested and homogenized in a Dounce homogenizer. Nuclei were removed by low-speed centrifugation. Virus containing supernatant was sonicated three times and then centrifuged through a sucrose cushion (36% sucrose; Carl Roth GmbH, Karlsruhe, Germany). Supernatant was discarded. The viral pellet was resuspended in Tris-HCl pH 9.0 (Sigma-Aldrich, Taufkirchen, Germany) and then sonicated again three times. Subsequently, virus was centrifuged through a continuous sucrose gradient (24–40%). The virus band was carefully harvested. The virus was centrifuged again and the pellet was resuspended in Tris-HCl pH 9.0. After three more sonication steps, the virus was stored in aliquots at −80 °C. Virus quantification was performed by titration on CV-1 cells.

### 3.3. Sulforhodamine B (SRB) Assay

Respective cell lines were seeded in 24-well plates and infected with GLV-1h94 at a multiplicity of infection of 0.1 (MOI 0.1). At 3 h post infection (3 hpi), the inoculum was removed and growth medium with or without 1 mmol/l of the prodrug 5-FC (Roche, Grenzach-Wyhlen, Germany) was added to the cells. At 96 hpi, the medium was removed and SRB assay was performed. As described previously [26], cells were washed using cold PBS and fixed with 10% trichloroacetic acid (TCA, Carl Roth GmbH, Karlsruhe, Germany) at 4 °C in a refrigerator for 30 min. TCA was removed and the wells were washed three times using tap water. Cell layers were dried at 40 °C for at least 6 h before staining with sulforhodamine B (SRB) staining solution (0.4% SRB in 1% acetic acid; Sigma-Aldrich, Taufkirchen, Germany) was performed at RT for 10 min. After washing the cells with 1% acetic acid and drying them, Tris(hydroxymethyl)aminomethane (Tris, 10 mM, pH 10.5) was added to each well in order to extract protein-bound dye and the plates were incubated at RT for 10 min. Optical density was measured in a microtiter plate reader (Tecan Genios Plus, Tecan Deutschland, Crailsheim, Germany) at a wavelength of 550 nm (reference wavelength at 620 nm).

### 3.4. Virus Growth Curves

To investigate the replication of GLV-1h94, the respective cell lines were seeded in 6-well plates and infected with an MOI of 0.1. Cells were harvested by scraping them into their medium at 3, 24, 48, 72, and 96 hpi. Three subsequent freeze/thaw cycles led to cell lysis and to the release of cell-bound viral particles. To assess the count of viral particles in the harvested suspensions, serial dilutions of the samples were titrated on CV-1 cells in 24-well plates as described previously [25]. After primary infection at 1 hpi, each well received overlay medium containing 1.5% carboxymethylcellulose (CMC, Sigma-Aldrich, Taufkirchen, Germany) and cells were further incubated for 2 days. Staining of the virus plaques was performed at RT by using crystal violet staining solution (Carl Roth GmbH, Karlsruhe, Germany) for 3 to 6 h. Subsequently, cells were washed with tap water, stained virus plaques were counted, and corresponding virus titers (plaque-forming units per milliliter (pfu/mL)) were calculated.

### 3.5. LC-MS/MS-Based Quantification of 5-Fluorocytosine and Its Metabolite 5-Fluorouracil

Cells were seeded in 6-well plates and infected with GLV-1h94 at an MOI of 0.01. At 3 hpi, the inoculum was removed and medium containing 0.1 mM 5-FC was added. Analyses were performed in triplicate. Supernatants were collected at 3 hpi, immediately after adding 5-FC, and at 24, 48, 72, and 96 hpi and incubated at 60 °C in a water bath for 30 min to inactivate the virus. Then, samples were snap-frozen in liquid nitrogen. For standard curves, cells were infected as described above. After inactivation of the virus, 5-FC and 5-FU were added to the supernatants at concentrations of 0, 0.01, 0.02, 0.04, 0.07, and 0.1 mM. For analysis, 100 µL methanol containing internal standard chlorouracil (corresponding to a final concentration of 0.05 mM; Sigma-Aldrich, St. Louis, MO, USA) were added to 400 µL sample or standard. Protein precipitation was performed by adding 800 µL acetonitrile. After 5 min of centrifugation at 13 000 g, 500 µL of the supernatants were evaporated at 60 °C in a vacuum evaporator and redissolved in 50 µL of 25% MeOH. Targeted quantification was achieved by injecting 2 µl sample extract into a 6500 QTRAP (Sciex, Darmstadt, Germany) mass spectrometer coupled to an Eksigent 200 microLC chromatography system (Sciex, Darmstadt, Germany). The autosampler was kept at 10 °C. For chromatographic separation, a BEH C18 column (1.0350 mm/1.7 µm; Waters, Milford, MA, USA) at 50 °C column temperature and a gradient of two mobile phases (A: water (LC-MS grade; Merck, Darmstadt, Germany) containing 0.2% formic acid(LC-MS grade; Fisher Chemical, Schwerte, Germany); B: acetonitrile (LC-MS grade; Honeywell, Charlotte, NC, USA) containing 0.2% formic acid) at a 30 µL/min flow rate were used. The percentage of solvent B was raised from initially 2% for the first 0.2 min to 10% in 2 min and to 80% solvent B in another minute. After column cleaning with 98% solvent B, the solvent composition was returned to the initial start conditions. The mass spectrometer was run in multiple reaction monitoring (MRM) mode using positive ion mode electrospray ionization (ESI). Source settings were set to 5 kV as ion spray voltage, 150 °C as source temperature, and a curtain gas of 30. Mass transitions (m/z) and compound-specific settings can be found in Table 1. Quality control (QC) samples in three concentrations (low level 0.015 mM, medium level 0.05 mM, and high level 0.075 mM) and two replicate calibration curves for quantification were included in each run. Data acquisition and quantification based on the calibration curve were performed using Analyst 1.6.3 (Sciex, Darmstadt, Germany).

## 4. Conclusions

Countless efforts aim to improve and optimize the treatment of cancer. In the early 19th century, observations were made that in many individual cases, tumor regression occurred with concurrent viral infections [27,28]. This phenomenon constituted the impetus for a new form of targeted cancer therapy, i.e., immunovirotherapy. Oncolytic viruses utilize methods to directly destroy cancer cells, which then induce a long-lasting systemic anti-tumoral immune response [29,30]. Furthermore, the insertion of distinct transgenes into viral genomes not only helps to increase tumor specificity of oncolytic viruses, thereby intensifying their anti-cancer efficacies, but also attenuates viral properties in a way to further minimize harm to healthy tissues [27].

In this study, the potential of recombinant vaccinia virus GLV-1h94 was examined, which was designed to express SCD, a prodrug-converting enzyme encoded by the *fcu1* gene [7]. It could be shown that solid tumor cell lines of the NCI-60 panel responded with different levels of cellular resistance to GLV-1h94-based virotherapy. However, this resistance can be overcome by using the virus-encoded SCD prodrug system. A detailed investigation suggested that the cytotoxic effect of converted 5-FU is directed either against tumor cells or against virus replication, depending on the balance between cell line-specific susceptibility to GLV-1h94-induced oncolysis and 5-FU sensitivity. These data can be groundbreaking for the precise evaluation of clinical studies investigating virotherapeutics incorporating prodrug systems for combined chemovirotherapy.

## Figures and Tables

**Table 1:** Mass transitions and compound-specific settings during quantitative analysis.

		Precursor Ion (*m/z*)	Product Ion (*m/z*)	Retention Time (min)	CE ^1^	CXP ^2^	DP ^3^
**5-Fluorocytosine**	Quantifier	130	113	1.1	25	15	61
	Qualifier	130	87	1.1	25	15	61
**5-Fluoro-uracil**	Quantifier	131	114	1.35	25	15	61
	Qualifier	131	58	1.35	35	15	61
**5-Chloro-uracil**	Internal standard	146	130	1.57	23	13	100

^1^ CE, collision energy; ^2^ CXP, cell exit potential; ^3^ DP, declustering potential.
